# Budget cap and pay-back model to control spending on medicines: A case study of Bulgaria

**DOI:** 10.3389/fpubh.2022.1011928

**Published:** 2022-11-11

**Authors:** Zornitsa Mitkova, Maria Dimitrova, Miglena Doneva, Konstantin Tachkov, Maria Kamusheva, Lyubomir Marinov, Nikolay Gerasimov, Dimitar Tcharaktchiev, Guenka Petrova

**Affiliations:** ^1^Department of Organization and Economy of Pharmacy, Faculty of Pharmacy, Medical University of Sofia, Sofia, Bulgaria; ^2^Department of Pharmacology, Pharmacotherapy and Toxicology, Faculty of Pharmacy, Medical University of Sofia, Sofia, Bulgaria; ^3^Medical College, Trakia University, Stara Zagora, Bulgaria; ^4^University Endocrinology Hospital, Medical University of Sofia, Sofia, Bulgaria

**Keywords:** budget cap, healthcare budget, medicines budget, overspending, paid back

## Abstract

Central and Eastern European countries (CEEC) have among the highest rates of increase in healthcare expenditure. External reference pricing, generics and biologics price capping, regressive scale for price setting, health technology assessment (HTA), and positive drug lists for reimbursed medicines are among the variety of implemented cost-containment measures aimed at reducing and controlling the rising cost for pharmaceuticals. The aim of our study was to analyze the influence of a recently introduced measure in Bulgaria—budget capping in terms of overall budget expenditure. A secondary goal was to analyze current and extrapolate future trends in the healthcare and pharmaceutical budget based on data from 2016 to 2021. The study is a retrospective, observational and prognostic, macroeconomic analysis of the National Health Insurance Fund's (NHIF) budget before (2016–2018) and after (2019–2021) the introduction of the new budget cap model. Subgroups analysis for each of the three new budget groups of medicines (group A: medicines for outpatient treatment, prescribed after approval by a committee of 3 specialists; group B: all other medicines out of group A; and group C: oncology and life-saving medicines out of group A) was also performed, and the data were extrapolated for the next 3 years. The Kruskal–Wallis test was applied to establish statistically significant differences between the groups. During 2016–2021, healthcare services and pharmaceutical spending increased permanently, observing a growth of 82 and 80%, respectively. The overall healthcare budget increased from European €1.8 billion to 3.3 billion. The subgroup analysis showed a similar trend for all three groups, with similar growth between them. The highest spending was observed in group C, which outpaced the others mainly due to the particular antineoplastic (chemotherapy) medicines included in it. The rising overall healthcare cost in Bulgaria (from European €1.8 billion to 3.3 billion) reveals that implementation of a mechanism for budget predictability and sustainability is needed. The introduced budget cap is a relatively effective measure, but the high level of overspending and pay-back amount (from European €34 billion to 59 billion during 2019–2021) reveals that the market environmental risk factors are not well foreseen and practically implemented.

## Introduction

In 1998, the World Health Organization (WHO) raised awareness of the fact that the growth of expenditure for medicines had outpaced the growth in the gross domestic product (GDP) of the world economy by four times (1). Subsequent research showed that per-person healthcare costs had grown by 2.3%, whereas GDP per person had increased by only 1.5% for the period 2000–2018 (2). The same report outlined that only eleven out of 52 countries had reported that GDP growth was higher than the growth in healthcare costs, whereas, in 31, the share of public expenditure had risen more than twice the GDP. The direct costs associated with non-communicable diseases (NCDs) are expected to grow by 0.8% per year in EU countries between 2014 and 2020, with the main factors leading to this being the aging EU population, as well as the introduction of new health technologies (3).

Post-soviet Central and Eastern European Countries (CEEC) have seen some of the highest rates of increases in healthcare expenditure. A possible reason for this could be the transition from planned to market economies, with the implementation of many new regulations leading to gaps between regulatory and control measures. Furthermore, the influx of new medicines could have introduced the need for faster endorsement of control measures pertaining to the pharmaceutical area, in order to control prices and reimbursement, as well as pharmaceutical spending (4).

Over the years, a variety of cost-containment approaches, aimed at controlling the rising cost of pharmaceuticals, have been employed; implementation of positive drug lists of all reimbursed medicines, establishment of regulatory bodies on prices and reimbursement, external reference pricing for manufacturer price setting, regressive scale for price setting, generics and biologics price capping, health technology assessment (HTA) for new medicines before inclusion in the positive drug list (PDL), discounts of medicinal products, and other financial-based managed entry agreements negotiated with marketing authorization holders (MAH) have continuously been introduced in the regulation of CEEC (5–7). Most of these measures are also introduced in Bulgaria (8) where they aim to control prescribers, producers, or the whole market, but their impact on the overall budget has not been defined.

In addition to the measures aimed at controlling the rising cost of medicines, other financial budget models were developed such as annuity (9), Netflix model (10), and a variety of forecasting models such as Andersen's behavior model (11, 12), micro-, component-based, and macro-models (13)to reduce and manage budget growth or help consumers (14).

In 2018, the Bulgarian National Health Insurance Fund (NHIF) (15) introduced a budget cap model for pharmaceuticals in order to control the growth of expenditure for pharmaceuticals by separating all reimbursed medicines into three groups, according to their contribution to the budget (group A: medicines for outpatient treatment, prescribed after approval by a committee of three specialists; group B: all other medicines out of group A; and group C: oncology and life-saving medicines out of group A). The maximum reimbursed budget for each group is negotiated with the marketing authorization holders four times annually. If the reimbursed budget in the group exceeds the negotiated cost, pharmaceutical companies return revenue respective to the proportion of their market share and budget increase above the negotiated value. The effectiveness of this measure has not been studied until now and that provoked our interest in the topic.

The aim of this study is to analyze the trends in the healthcare and pharmaceutical budget for the period 2016–2021 and to forecast future tendencies. In addition, we also performed a subgroup analysis of the three budget groups of medicines for the period 2019–2021, after the introduction of the new model.

The main question we wanted to answer was whether the rate of growth of the budget decreased after the introduction of the new model.

## Methods

### Design of the study

The study is a retrospective, observational and prognostic, macroeconomic analysis of the NHIF budget for healthcare services and medicines during 2016–2021. The spending information for healthcare services and medicines was extracted from official sources and compared for both periods. The first period encompasses the time before the introduction of the new budget cap model (2016–2018) and the second one, after that (2019–2021).

The data included in the analysis were selected from different sections of the NHIF webpage. As the officially published information was unstructured, we used a four-step approach to systematize it. First, we identified NHIF codes of medicines considering each individual trademark and respective reimbursed expenditure. Second, all trademarks were systematized according to the International Nonproprietary Names (INNs) of medicines and arranged into the main financial groups (A, B, and C), according to NHIF requirements for budget predictability. Third, we calculated the reimbursed expenditure and annual reimbursed spending for each year of consideration and time period for every INN. Fourth, we extracted the information for the overspending of the medicines per budget cap groups (A, B, and C) and summarized it for each year.

Subgroup analysis was also conducted for each of the new budget groups of medicines (A, B, and C) by pharmaco-therapeutic and ATC groups in order to explore which medicines have the highest contribution to the budget growth. We extrapolated the budget data for the next 3 years for every subgroup, calculated the share of the budget increase, and compared those shares.

### Data sources

Healthcare and pharmaceutical spending data during 2016–2021 were collected from the official government newspapers approved by the parliament.

Information about the real pharmaceuticals' expenditure and the pay-back sums was collected from the NHIF database for every subgroup of medicines. For the subgroup analysis, the officially published information covers several packages and reimbursed amounts for each budget group of medicines (groups A, B, and C) including pharmaco-therapeutic groups (16).

All costs are presented in Euro at the fixed exchange rate of 1 Euro = 0.51 BGN.

### Quantitative analyses

For data analysis, we employed the following quantitative and statistical methods: an index analysis, extrapolation based on time series analysis, and Kruskal–Wallis test.

Indexes of budget change were calculated using two approaches. The first one is as a chain index where the spending each year is divided by the previous year's spending (2017/2016 year; 2018/2019 year; 2020/2019 year; 2021/2020 year). The second is as a basic index where the first year in the observed period, namely 2016, is taken as a base and each year is divided by the base year (17, 18). The chain and basic indexes illustrate two different points of view—the rate of difference each year compared to the previous one, and the rate of change in each year compared with the first year of observation. In this way, differences by period can be examined over a wide range, and the most significant changes can be assessed.

The Kruskal–Wallis test was applied to establish statistically significant differences between public spending during the observed period and to compare proportions (19). It is a non-parametric method used for the comparison of independent samples. We consider it the most appropriate test to assess reimbursed spending and potential statistically significant differences between applied chain and basic indexes because they are not normally distributed. Med Calc vers.16.4.3 (Ostend, Belgium; 2016) software was applied.

The final calculative method was an extrapolation based on the principles of time series analysis (20). We used it to determine the probable values of the future reimbursed sales based on the time trends. We apply the extrapolation of sales data for the next 3 years (2022–2024) based on data for the previous 3 years to be more precise and match the same time period of observation and future reimbursed sales. This way we can illustrate the current and expected trend of reimbursed amounts considering the main group of medicines (groups A, B, and C).

## Results

### Budget analysis

**Figure 1:**
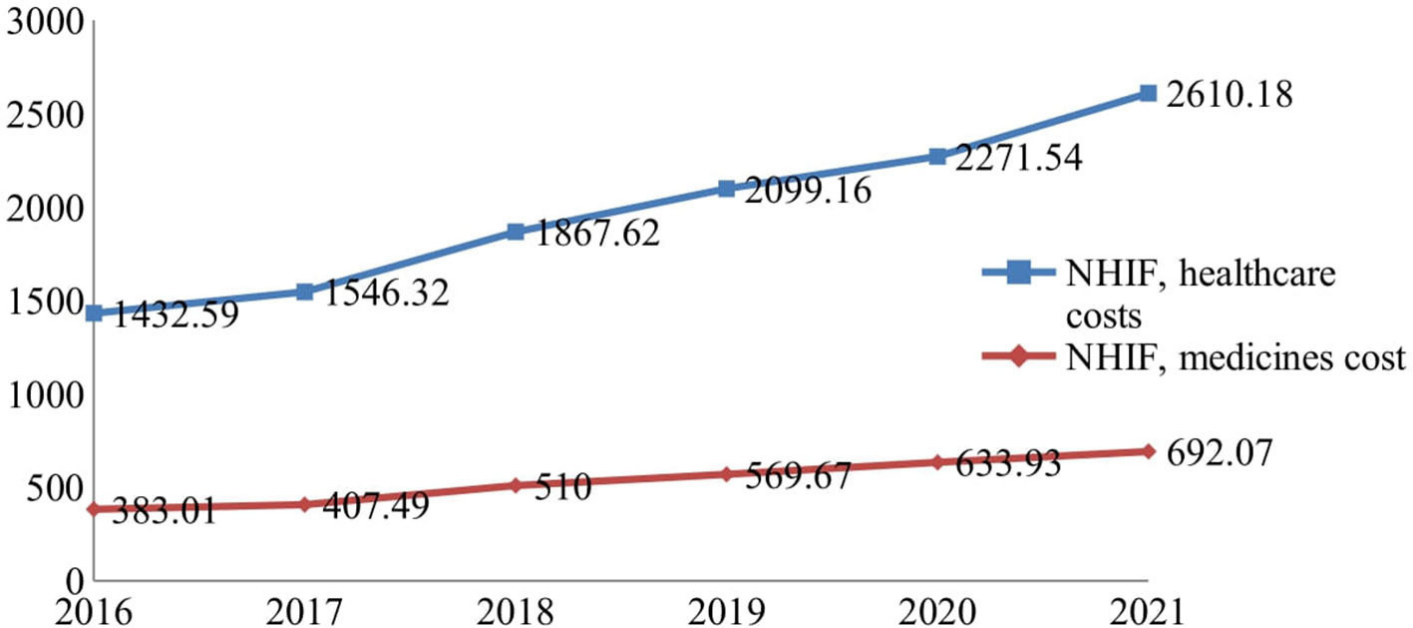
National Health Insurance Fund (NHIF) budget for healthcare services and medicines (Euro, mln).

During 2016–2021, healthcare services and pharmaceutical spending increased permanently, observing a growth of 82 and 80%, respectively, at the end of the period (Figure 1).

The total healthcare budget rose from European €1.8 billion to 3.3 billion, and the relative contribution of pharmaceuticals was on average 21.25 ± 0.36%. The average index of budget increase for medicines is 1.126 ± 0.73 vs. 1.128 ± 0.054 for healthcare costs. The indexes vary from 1.06 to 1.25 (Table 1). Comparing the indexes during the first 3 years (2016–2018) with that of the second 3-year period (2019–2021), we observe a decrease in the values of these indexes and their growth despite the permanent increase in the budget for medicines.

**Table 1:** Healthcare services and pharmaceuticals budget growth (mln Euro).

**Exploring parameter**	**2016**	**index**	**2017**	**Index**	**2018**	**Index**	**2019**	**Index**	**2020**	**Index**	**2021**
NHIF, healthcare services costs—chain index^*^	1432.59	1.08	1546.32	1.21	1867.62	1.12	2099	1.08	2271.54	1.15	2610.18
NHIF, medicines cost chain index^*^	383.01	1.06	407.49	1.25	510	1.12	570	1.11	633.93	1.09	692.07
NHIF, medicines cost basic index^**^		1.06		1.33		1.48		1.65		1.81	
Total healthcare costs and basic indexes^**^	1815.60	1.07	1953.81	1.31	2377.62	1.47	2669	1.60	2905.47	1.82	3302.25
Medicines as part of total budget (%)	21.10		20.86		21.45		21.35		21.82		20.96

* chain indexes calculated between each two subsequent years.

** fixed base indexes calculated by dividing each year to 2016.

On average, the budget growth for healthcare services cost and medicine are 3865.16 ± 875.23 and 1044.5 ± 241.13, respectively. The Kruskal–Wallis test revealed statistically significant differences between all compared indexes (*p* < 0.0001).

### Subgroup cost analysis for medicines after budget cap introduction

After extracting the costs from the real expenditure for medicines, we can see that there is budget overspending and the expenditure is higher than the projected cost (Tables 2, 3).

**Table 2:** Annual NHIF spending and rate of index of change during 2019–2021 (Euro).

	**Expenditure 2019**	**Index 2020 vs. 2019**	**Expenditure 2020**	**Index 2021 vs. 2020**	**Expenditure 2021**
Group A	222,755,603	1.12	250,547,170	1.06	266,210,908
Group B	151,593,119	1.04	157,993,953	1.01	159,448,726
Group C	234,275,772	1.26	294,042,467	1.13	331,015,921
Total expenditure, Euro	608,624,495	1.15	702,583,589	1.08	756,675,555

**Table 3:** Paid-back expenditure by Marketing Authorisation Holder (MAH).

**Budget group of medicines**	**2019**	**2020**	**2021**
	**Exceeding amount, Euro**	**Exceeding amount, Euro**	**Exceeding amount, Euro**
Group A	902,627.07	22,559,466.48	15,800,907.72
Group B	1,131,246.30	4,245,937.68	3,482,188.2
Group C	32,188,504.7	46,829,660.64	39,805,921.26

There is a budget growth for the whole period as it is most evident in group C with declining indexes. Therefore, group C has the highest contributing rate to expenditure increase.

The new budget model is based on negotiation with the companies for the cap value of the expenditure and, in case of budget drilling, the companies pay back the exceeded sum. The accepted model has led to the overall payback into the budget of European €34 million in 2019 to European €59 million in 2021. Group C is once again with the highest payback amount, but in 2021, the sum that was returned had decreased. It is also evident that the payback is lower than the overspending. The negotiated payback is not publicly revealed, and we cannot discuss who covers the rest of the expenditure but it is highly likely that it is the NHIF (Table 3).

The subgroup analysis shows that antineoplastic medicines contribute with the highest rate toward the expenditure; nevertheless, they are distributed in two budget groups, followed by antidiabetic medicines (Table 4). The latter corresponds with the morbidity patterns in the country and areas of the faster introduction of new technologies.

**Table 4:** Pharmaco-therapeutic groups with the highest reimbursed spending during 2019–2021.

	**Pharmaco- therapeutic groups (ATC code)**	**Reimbursed spending paid by NHIF, Euro**		
		**2019**	**2020**	**2021**
**Group A**	Antineoplastic and immune modulating agents *(ATC L01, L02, L03, L04)*	92,618,969.22	100,352,463.87	105,163,926.78
	Medicines used in diabetes (*ATC A10)*	52,561,598.07	60,354,025.26	62,061,145.20
	Anti-infective for systemic use *(ATC J01)*	34,473,807.51	-	
	Nervous system (*ATC N03, N04, N05, N06,N07)*		31,718,183.67	33,065,438.43
**Group B**	Respiratory system (*ATC R03, R05)*	38,670,312.42	39,505,463.43	38,492,289.27
	Cardiovascular system *(ATC C01, C02, C03, C04, C07, C08, C09, C10)*	36,646,981.26	54,430,917.39	35,725,513.26
	Antithrombotic agents (*ATC B01)*	29,984,085.24	33,962,781.90	37,680,754.32
**Group C**	Antineoplastic agents (*ATC L01, L02)*	247,092,784.05	312,641,611.68	352,789,198.26
	Blood and blood forming organs*(ATC B02, B03)*	6,365,626.20	7,029,557.46	8,200,311.42
	Drugs affecting bone structure and mineralization (*ATC M05)*	5,839,378.62	6,793,392.27	7,714,503.78

No statistically significant differences were found comparing reimbursed spending paid by the NHIF for the latest 3 years *via* Kruskal–Wallis test (*p* = 0.886).

### Expenditures forecast

**Figure 2:**
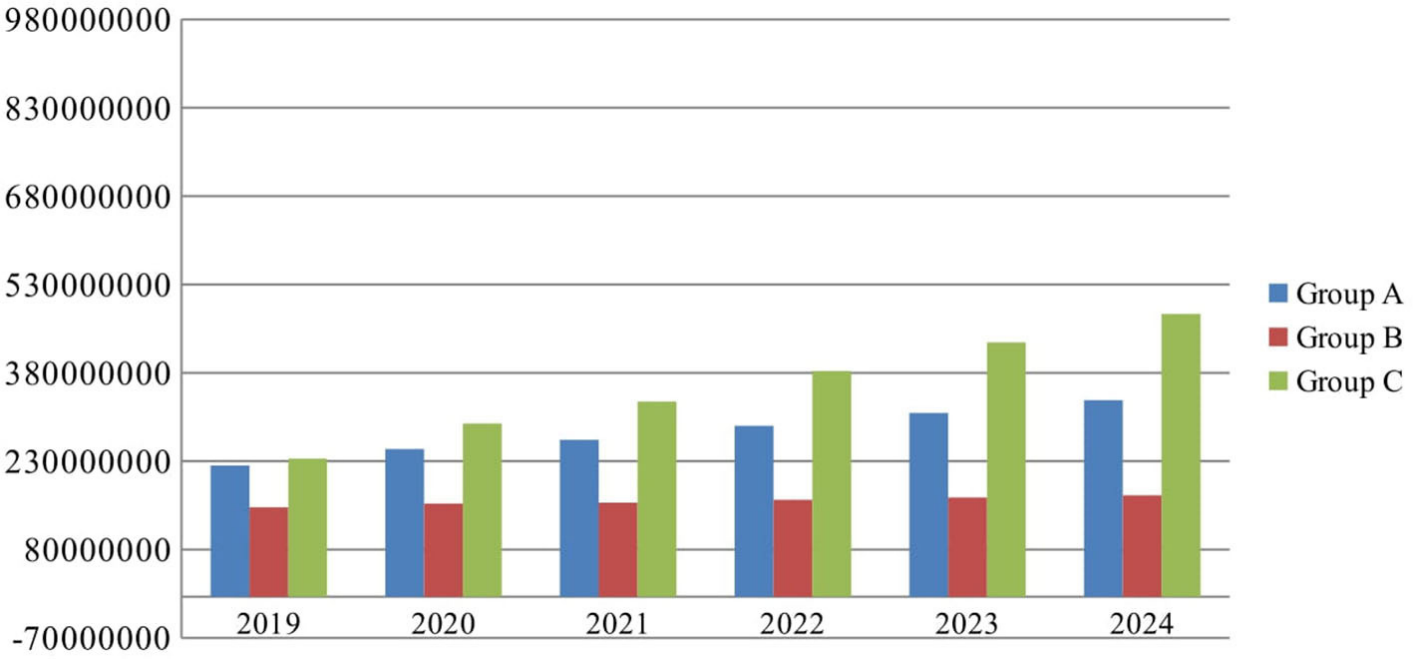
Expenditures forecast for 2022–2024.

Based on the current rising levels, we extrapolated the public expenditure for medicines for the next 3 years, in order to check whether the budget cap model will continue to control the budget growth (Figure 2).

Logically, the expenditure for medicines is expected to increase, based on the extrapolation of the current trend, eventually reaching around European €480 million by the end of 2024. Group C will continue to be the main cost driver, followed by group A, while group B is expected to stay at a constant value.

## Discussion

Bulgaria currently has the lowest per capita expenditure for healthcare out of all EU countries, both in absolute terms and as a share of GDP (21). Previous comparisons of macroeconomic and healthcare spending between Balkan and Eastern European countries from 1995 to 2014 revealed the biggest growth in Bulgaria, Serbia, and Slovenia. The largest median spending on health as a percentage of GDP was found in Bosnia and Herzegovina and Greece, and the smallest one was found in Turkey and Romania (22). A previous study confirmed that the main factor, among others, leading to increased healthcare costs in Bulgaria was the increase in GDP (23).

The budget cap and co-payment policies can reduce the utilization of medicines and create some savings in the short-term period. At the same time, decreasing the consumption of the life-saving group of medicinal products and those used for chronic diseases could impact negatively patients and healthcare costs, resulting in increasing payment for hospital treatment (24). The assessment of budget cap policy impact on healthcare spending in the long-term period depends on a variety of factors as well as the design and methodology. Budget cap design requires considering disease prevalence and rate of inflation. The active monitoring of new technologies and their high costs could be incorporated into budget planning as some specific conditions may require additional costs (25). Italy has a similar model of managed entry agreements (MEAs), an analysis of which also revealed a discrepancy between expected payback and collected payback. The calculated total theoretical payback was estimated at €46.3 mln in 2013, but only 31.3 mln was collected. It is worth noting that the Italian system of pharmaceutical expenditure control is based on two main categories of medicines [essential drugs and drugs for chronic diseases (class A) and medicines for hospital utilization (H)] limited to various ceilings which are paybacks in case of overspending (26, 27). Other implemented price-volume schemes, volume of sales related to a target population, confidential discounts, and payback schemes are commented on in Poland and Hungary. According to the authors, this policy tool allows rational spending, while ensuring patient access to new medicines (28).

To the best of our knowledge, this is the first national study exploring budget tendencies after the budget cap with the pay-back model was introduced. The study findings illustrated rising reimbursed spending for medicines in Bulgaria after 2018. The time series analysis is applied as a forecasting approach for price impact examination, results of new regulation, and medicines utilization analysis. It allows discussion on prognostic data in short- and long-term periods (19, 29). The inclusion of new medicines in the PDL, large chronic disease spread, rate of inflation, and rising GDP altogether affect public expenditure for medicines in Bulgaria. At the same time, we found that budget capping has nonetheless introduced a measure of control over the growth of the budget, illustrated by the calculated indexes and their different rates of increase pre- and post-2018. The high value of revenue paid back by MAH indicates that not all factors contributing to the budget increase are incorporated in the annual budget planning. In this respect, the COVID-19 pandemic led to increased public spending on healthcare resources and pharmaceuticals and could be considered an important cost driver.

A recently introduced budget cap model in Spain links pharmaceutical spending to GDP. A report indicated that pharmaceutical cost control through that methodology is not effective, and it is inadequate when considering entry and diffusion of innovation (30).

When the medicine, spending ceiling is exceeded in Greece, the companies return revenue above that as a direct cash return to National Organisation for Health Services (EOPYY, Eθνικóς Oργανισμóς Παρoχνς Yπηρεσιων Yγεíας). The Greek budget cap was introduced as a temporary measure and was linked to real GDP growth and implemented as a claw-back mechanism. It resulted in lower medical service and pharmaceutical expenditures but also some delay due to the complexity associated when expenditures exceed the ceiling (31). The introduction of budgetary targets improves cost allocation and cost-benefit considerations. Participation of all stakeholders along with analysis of age-related morbidity and medical progress spending prediction could minimize the overall risk and support the implementation of effective measures (32).

## Conclusion

Our study shows that the introduction of the budget cap model by separating the medicines into three main groups allows for budget predictability in the face of continuously rising expenditures. The main pharmaco-therapeutic groups with the highest contribution to the costs are those with the most expensive new technologies (antineoplastic and immunomodulatory agents, drugs affecting bone structure and mineralization) and those covering diseases with the largest prevalence in Bulgaria (nervous system, respiratory disease, diabetes, and cardiovascular diseases).

The limitation of our study is the lack of officially published data for the real sums returned by the industry. The other limitation is the fact that healthcare spending due to SARS-COVID-19 is not selected and categorized as a part of overall expenditure, despite this, spending indexes remained similar throughout the years. The inflation rate in healthcare is between 0.2 and 1.3% within the study period which could also be the reason for rising healthcare services expenditure (33). We do not explore the consumer price indexes and inflation rate due to the following reasons. First, our analysis focuses on the whole budget and not on individual items. Second, during the observed period, inflation was relatively stable due to the fixed exchange rate of the currencies. Further studies are needed to explore the trends in healthcare costs in a long-term period after the budget cap and pay-back model implementation as well as price index changes due to the inflation rate.

The rising overall healthcare cost in Bulgaria (from European €1.8 billion to 3.3 billion) reveals that implementation of a mechanism for budget predictability and sustainability was needed. Moreover, the extrapolation of reimbursed spending suggests that an increase for the next 3-year period is expected, thus reaching European €985 million in 2024. The introduced budget cap is a relatively effective measure, but the high level of overspending and pay-back amount (from European €34 billion to 59 billion during 2019–2021) reveals that the market environmental risk factors are not well foreseen and practically implemented.

## Data availability statement

The raw data supporting the conclusions of this article will be made available by the authors, without undue reservation.

## Author contributions

GP conceived and designed the investigation. ZM collected the data and performed the results section. MDi and MK wrote the introduction and conclusion. KT and NG wrote the discussion. KT and MDo prepared the statistical analysis. DT and LM wrote the methodology, result draft, and validated statistics. All authors wrote and revised the manuscript and approved its submission for publication, confirming that the study is original.

## Funding

This study is part of the National Scientific Program Electronic Healthcare in Bulgaria (E-health), supported by the Ministry of Education and Science.

## Conflict of interest

The authors declare that the research was conducted in the absence of any commercial or financial relationships that could be construed as a potential conflict of interest.

## Publisher's note

All claims expressed in this article are solely those of the authors and do not necessarily represent those of their affiliated organizations, or those of the publisher, the editors and the reviewers. Any product that may be evaluated in this article, or claim that may be made by its manufacturer, is not guaranteed or endorsed by the publisher.
